# Optimal Laboratory Cultivation Conditions of *Limnospira maxima* for Large-Scale Production

**DOI:** 10.3390/biology12121462

**Published:** 2023-11-24

**Authors:** Yirlis Yadeth Pineda-Rodríguez, Diana Sofia Herazo-Cárdenas, Adriana Vallejo-Isaza, Marcelo F. Pompelli, Alfredo Jarma-Orozco, Juan de Dios Jaraba-Navas, Jhony David Cordero-Ocampo, Marianella González-Berrio, Daniela Vegliante Arrieta, Ana Pico-González, Anthony Ariza-González, Katia Aviña-Padilla, Luis Alfonso Rodríguez-Páez

**Affiliations:** 1 Departamento de Ingeniería Agronómica y Desarrollo Rural, Maestría en Ciencias Agronómicas, Facultad de Ciencias Agrícolas, Universidad de Córdoba, Montería 230002, Colombia; dvegliantearrieta41@correo.unicordoba.edu.co (D.V.A.); apicogonzalez@correo.unicordoba.edu.co (A.P.-G.); aarizagonzalez28@correo.unicordoba.edu.co (A.A.-G.); 2 Laboratorio de Sanidad Acuícola y Calidad de Agua, Facultad de Medicina Veterinaria y Zootecnia, Universidad de Córdoba, Montería 230002, Colombia; dherazo@correo.unicordoba.edu.co (D.S.H.-C.); advalis3@gmail.com (A.V.-I.); 3 Laboratorio de Biología Molecular Aplicada, Facultad de Ciencias Agrícolas, Universidad de Córdoba, Montería 230002, Colombia; ajarma@correo.unicordoba.edu.co (A.J.-O.); jjaraba@correo.unicordoba.edu.co (J.d.D.J.-N.); larguez@fca.edu.co (L.A.R.-P.); 4 Departamento de Ciencias Acuícolas, Programa de Acuicultura, Facultad de Medicina Veterinaria y Zootecnia, Universidad de Córdoba, Montería 230002, Colombia; jcorderoocampo82@correo.unicordoba.edu.co (J.D.C.-O.); mgonzalezberrio@correo.unicordoba.edu.co (M.G.-B.); 5 Centro de Investigación y de Estudios Avanzados del I.P.N. Unidad Irapuato, Irapuato 36821, Mexico; ib.katia@gmail.com

**Keywords:** nitrogen source, food security, cyanobacteria, *Arthrospira maxima*, phycobiliproteins, biomass production

## Abstract

**Simple Summary:**

*Limnospira maxima*, a photosynthetic bacterium with valuable proteins and pigments, faces cultivation challenges due to a lack of management knowledge and expensive nitrogen sources. This study aimed to find an efficient method for large-scale cultivation through testing different light colors and nitrogen sources. The study’s results showed that using white lights and potassium nitrate was the most effective approach, promoting growth and increasing the production of phycocyanin, a valuable blue pigment with applications in food, health, and the dye and pigment industry. In summary, this research offers a cost-effective and efficient solution for cultivating *Limnospira maxima* on a larger scale. This has the potential to positively impact food production and various industrial goods, providing a more sustainable and accessible source of phycocyanin and other valuable components.

**Abstract:**

Cultivating *Limnospira maxima*, renowned for its abundant proteins and valuable pigments, faces substantial challenges rooted in the limited understanding of its optimal growth parameters, associated high costs, and constraints in the procurement of traditional nitrogen sources, particularly NaNO_3_. To overcome these challenges, we conducted a comprehensive 4 × 3 factorial design study. Factors considered included white, red, blue, and yellow light spectra, along with nitrogen sources NaNO_3_ and KNO_3_, as well as a nitrogen-free control, for large-scale implementation. Optimal growth, measured by Optical Density, occurred with white and yellow light combined with KNO_3_ as the nitrogen source. These conditions also increased dry weight and Chl-a content. Cultures with nitrogen deprivation exhibited high values for these variables, attributed to carbon accumulation in response to nitrogen scarcity. Phycocyanin, a crucial pigment for nutrition and industry, reached its highest levels in cultures exposed to white light and supplemented with KNO_3_, with an impressive content of 384.11 g kg^−1^ of dry weight. These results highlight the efficacy and cost-efficiency of using a combination of white light and KNO_3_ for large-scale *L. maxima* cultivation. This strategy offers promising opportunities to address global food security challenges and enhance the production of industrially relevant pigments.

## 1. Introduction

The persistence of global hunger poses a significant challenge, especially in developing countries. Ensuring the availability of and access to food remains a vital goal [1]. However, this concern is exacerbated by various factors, including population growth, economic disparities, and limited arable land. In recent years, the COVID-19 pandemic has had a detrimental impact on efforts to combat hunger, increasing undernourishment from 8.0% to 9.8% between 2019 and 2021. This translates to a 31.9% increase in women and a 27.6% increase in men experiencing food insecurity. Additionally, an estimated 22% of children under the age of five suffer from stunted growth, 6.7% experience wasting, and 5.7% are overweight. Approximately 3.1 billion people lack access to a healthy diet due to inflation stemming from this global crisis and the measures taken to address it [2]. The cultivation of Spirulina (*Limnospira maxima*), also known as *Arthrospira maxima*, emerges as a promising solution to address this food crisis due to its exceptional nutritional value. This is evidenced by its high protein content, representing 60–70% of its dry weight, and its vitamin B_12_ content. Around 60% of its protein content consists of phycobiliproteins, highly valued pigments in the pharmaceutical, nutraceutical, cosmetic, and food industries [3,4,5]. These qualities have promoted its trade and consumption by millions of people worldwide, endorsed by governments, health agencies, and associations from more than 60 countries [6]. The United Nations Food Conference designated it as the food of the future, the World Health Organization (WHO) described it as the optimal product for human health [7], and it has been labeled a “superfood”. Even NASA recognizes its potential as a source of sustenance in space travel due to its exceptional nutrient density [8]. Biomass is commonly consumed in the form of nutritional supplements, whether in dry powder, flakes, or capsules [9]. Another significant feature of *L. maxima* cultivation is its short reproductive cycle and the ability to thrive in resource- and space-constrained areas [10]. Being widely cultivated, it contributes to over 30% of global microalgal biomass production [4,11], generating approximately 20,000 metric tons annually [12]. However, its potential is constrained by a lack of knowledge regarding its optimal cultivation conditions and the high costs and limited availability of its traditional nitrogen source, sodium nitrate (NaNO_3_), which costs approximately USD 100 to USD 170 per kg. In contrast, the alternative nitrogen source, potassium nitrate, costs between USD 2 and USD 4 per kg. In order to address these limitations and harness the potential of *L. maxima* as a food resource, our main objective was to comprehensively evaluate different light spectra and nitrogen sources in the cultivation of *L. maxima* for large-scale implementation. In particular, alternative nitrogen sources such as KNO_3_ and light conditions for the cultivation of this cyanobacterium were explored to boost both food production and the pigment industry. In previous studies, Mousavi and colleagues [13] found that culture media supplemented with NaNO_3_ and KNO_3_ resulted in the highest biomass production (~1.18 g L^−1^). Other studies have emphasized the importance of light in pigment production, especially phycobiliproteins [14,15,16]. This is demonstrated in the study conducted by Bachchhav et al. [17], who found that spirulina cultivated with yellow LED lighting under mixotrophic conditions achieved the highest phycocyanin content (380 mg g^−1^ of dry biomass), while the study by Park and Dinh [15] found that red LEDs and white fluorescent light promoted higher growth and chlorophyll a (Chl a) concentration, whereas blue LEDs increased phycobiliprotein levels. Lastly, this research aimed to enhance the efficiency and productivity of *Limnospira maxima* cultivation to address global food security, especially in vulnerable regions. Furthermore, it contributed to the development of a sustainable source of nutrients and pigments with valuable industrial applications and promoted sustainability in food production through exploring innovative cultivation practices that reduced reliance on expensive and scarce nitrogen sources.

## 2. Materials and Methods

### 2.1. Cyanobacteria Strain and Production Medium

The strain *Limnospira maxima* was generously provided by AGROIMSA—Mexico as part of the reciprocal cooperation agreement MVZ—002-2015. Registered under OR195505.1 in GenBank [18], this strain has been acclimatized to laboratory conditions and cultured using a modified Zarrouk medium [19]. The research was conducted at the Aquaculture Health and Water Quality Laboratory of the University of Cordoba (Montería, Colombia; 8°47′37″ N; 75°51′51″ W, 15 m a.s.l.). Culture containers of 4 L capacity, containing 3 L of medium, were employed. The composition comprised 20% of the modified Zarrouk culture medium [19] infused with a nitrogen source (NaNO_3_, KNO_3_, or control—without nitrogen, WN), 20% inoculum of *L. maxima* (~1.2 g/L), and 60% deionized water supplemented with 1 g L^−1^ sea salt. To achieve the appropriate pH levels, NaOH, KOH, and Ca(OH)_2_ were utilized for the NaNO_3_, KNO_3_, and WN treatments, respectively. Continuous aeration was maintained in all experimental units, supplied using an aquarium pump (Aquarium pump, PUMPOWER^®^, model PR-3710, Dublin, Ireland).

The study adopted a completely randomized design with a 4 × 3 factorial arrangement, incorporating 4 irradiation sources (white, red, blue, and yellow), 3 nitrogen sources (NaNO_3_, KNO_3_, and WN), and 5 repetitions (Supplementary Figure S1). The illumination was provided continuously from one side of the Culture containers by using 18 W LED lamps of 120 cm. Further details on the lamps and their emitted photosynthetically active radiation (PAR) are presented in Table 1. Within each plastic container, a 50 × 50 mm photovoltaic cell, regulated using a C and C++ Arduino Uno R3 Microcontroller A000066 (Arduino Inc., Belmont, CA, USA; accessed on 13 June 2023), was utilized to monitor the PAR received by each culture medium at the cyanobacterial level. Measurements were recorded at two-hour intervals. The cultures were maintained under controlled conditions of temperature (24 ± 3 °C) and a photoperiod of 12 h light/12 h darkness, for a total of 27 days.

### 2.2. Optical Density and Growth Rate—Biomass

The evaluation of growth rate relied on measuring the optical density (OD) at 680 nm using a microplate reader (ThermoScientific™ Multiskan™ GO, Missouri City, TX, USA). The measurement process consisted of taking 1.5 mL of the culture medium for optical density measurement and 5 mL for dry weight determination. For dry weight assessment, the culture medium was vacuum-filtered using 47 mm diameter 0.5 μm Whatman^®^ membrane filters (part number WHA7585004, Sigma-Aldrich, Darmstadt, Germany) that were pre-dried in an oven (70 °C for 24 h) and weighed on an analytical balance (Sartorius Analytical Balance mod. ENTRIS224-1S, Bradford, MA, USA) with a precision of 0.1 mg. The filtered biomass was subsequently washed with distilled water to remove excess salts, dried in an oven at 70 °C for 24 h, subjected to a desiccator for 1 h, and re-weighed. The dry weight was determined through calculating the difference between the initial and final weights of the filter with the biomass, divided by the volume of the filtered sample, and expressed in g L^−^^1^. Both optical density (OD) and dry weight (DW) were measured at intervals of 3 days, spanning up to the 27th day of the experiment.

### 2.3. Biochemical Analysis

#### 2.3.1. Chlorophyll “a” and Total Carotenoids

The determination of chlorophyll content was conducted following the method outlined by Wellburn [20], with adaptations to facilitate measurement using a microplate reader. Every 3 days, a 10 mL aliquot of the culture was extracted and rapidly frozen at −65 °C for a minimum of 18 h. After thawing the tubes containing 10 mL of culture at 25 °C, they were centrifuged at 25 °C and 5000× *g* for 10 min. The supernatant was then discarded, and the pellet was used for chlorophyll extraction. To each 2 mL polypropylene tube (Sigma-Aldrich, Darmstadt, Germany, part number Z760951), pre-prepared with 0.1% poly(vinylpolypyrrolidone) (Sigma-Aldrich, part number 77627), 2 mL of 99% methanol (Sigma-Aldrich, part number 179337) was added. The tubes were mixed for 30 s, enveloped in aluminum foil, and placed in a thermo-shaker (Multitherm, Benchmark Scientific, Sayreville, NJ, USA) for incubation at 80 °C and 500 rpm for 5 min. Afterward, the tubes were centrifuged at 4 °C for 5 min at 15,000× *g*. The supernatants were transferred to new microtubes for chlorophyll measurement, while the pellets were promptly frozen at −65 °C to measure soluble proteins. For chlorophyll analysis, 200 μL of the extracted solution was dispensed into a glass microplate and read using a microplate reader (ThermoScientific™ Multiskan™ GO, Missouri City, TX, USA). Absorbance was recorded at 652.4 nm, 665.2 nm, 470 nm, and 720 nm (as a negative control). The concentrations of chlorophyll “a” (Chla) and total carotenoids (Car) were estimated using the equations proposed by Lichtenthaler and Buschmann [21].

#### 2.3.2. Phycobiliprotein Measurement

Phycobiliproteins were quantified following the method proposed by Bennett and Bogorad [22], with adaptations for the current research. Samples of 10 mL of the *L. maxima* cell suspension were extracted and vacuum-filtered as previously described. The filtered biomass was re-suspended in 2 mL of a 100 mM pH 7.4 phosphate buffer (Sigma-Aldrich, part number P5244) and briefly mixed for 5 s. The resuspended samples were then shielded from light, frozen at −40 °C for 24 h, and maintained at 4 °C until the appearance of the blue pigment (~24 h). Once pigment extraction was complete, the samples were kept chilled to prevent temperature-induced degradation, followed by centrifugation at 15,000× *g*. The resulting supernatant was used to quantitatively assess all phycobiliproteins spectrophotometrically, as per the equations outlined by Bennett and Bogorad [22].

#### 2.3.3. Soluble Proteins Measurement

For the determination of soluble proteins, the pellet retained from the chlorophyll analysis serves as the starting point. To extract soluble proteins, the pellet undergoes a series of washes using 70% ethanol (Sigma-Aldrich, part number 493511), with each washing step performed 3 to 4 times. During each wash cycle, the pellet is thoroughly mixed and subsequently centrifuged at 4 °C at 13,000× *g* for 10 min. After discarding the supernatant, the pellet remains for subsequent protein measurements.

Following this, the pellet is treated with 400 μL of 0.1 M NaOH. The mixture is thoroughly mixed and then incubated at 95 °C for 1 h at 800 rpm. Subsequently, the tubes are centrifuged at 4 °C and 13,000× *g* for 5 min. After establishing a standard curve using Bovine Serum Albumin (BSA) as per the manufacturer’s instructions, the samples are prepared accordingly. The Protein Assay Dye Reagent Concentrate (Bio-Rad, Hercules, CA, USA, part number #5000006) was utilized in accordance with the manufacturer’s guidelines for protein quantification.

### 2.4. Experimental Design and Statistical Analysis

All data were subjected to two-way repeated measures ANOVA, with time as the dependent variable. This analysis was performed using SigmaPlot for Windows v. 14.0 (Systat Software, Inc., San Jose, CA, USA). Furthermore, to elucidate the relationships between the analyzed features, principal component analysis (PCA) was conducted through multivariate analysis. The PCA was performed using Minitab 18.1 (Minitab, Inc., Chicago, IL, USA).

## 3. Results

### 3.1. Photosynthetically Active Radiation Measurement

As shown in Table 1, all light regimes exhibited consistent photosynthetically active radiation (PAR) levels across all treatments, with a PAR ranging from 77.51 ± 2.23 μmol photons m^−2^ s^−1^ under red LEDs to 79.31 ± 1.85 μmol photons m^−2^ s^−1^ under yellow LEDs. No significant differences in PAR were observed among the different media (*p* = 0.162). This uniformity ensures that all experimental units received equivalent PAR levels, differing only in terms of wavelength.

### 3.2. Optical Density (OD) and Growth Rate (DW)

**Figure 1:**
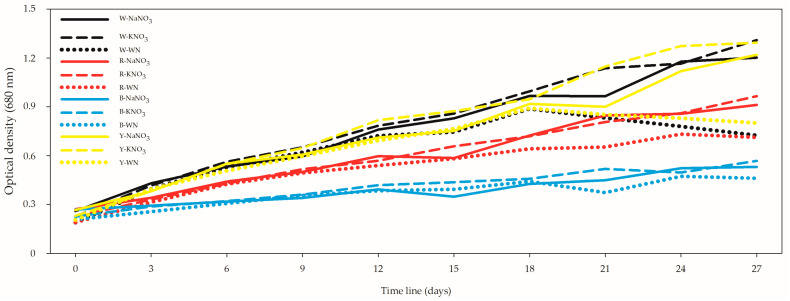
Effect of light spectra and nitrogen source on optical density of *Limnospira maxima* cultivated under white, red, blue, and yellow light spectra, supplemented with NaNO_3_, KNO_3_, and a control (WN). The values presented in the figure represent the means (±standard error) of optical density. When focusing exclusively on the 27-day *Limnospira maxima* cell suspensions, the most favorable results were achieved under both white and yellow LEDs. In the assessment of nitrogen sources, it was observed that both KNO_3_ and NaNO_3_ yielded comparable performance, with no statistically significant differences between them.

**Figure 2:**
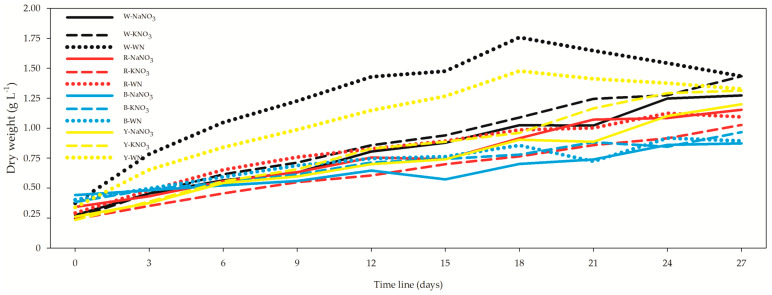
Impact of light spectra and nitrogen source on dry weight of *Limnospira maxima* cultivated under white, red, blue, and yellow light spectra, supplemented with NaNO_3_, KNO_3_, and a control (WN). The values presented in the figure represent the means (±standard deviation) of dry weight. Dry weight trends mirror those observed for optical density (OD), underscoring the superiority of white and yellow LEDs. Remarkably, over the 27-day period, the W-WN condition demonstrated a significant.

The optical density (OD) exhibited a range from 0.19 ± 0.01 in the R-WN *L. maxima* cell suspension on day 0 to 1.31 ± 0.02 in the W-KNO_3_ *L. maxima* cell suspension on day 27 (Figure 1). Meanwhile, the growth rate spanned from 0.23 ± 0.01 g L^−1^ in the Y-KNO_3_ *L. maxima* on day 0 to 1.76 ± 0.06 g L^−1^ in the W-WN *L. maxima* on day 18 (Figure 2). Upon comparing only the 27-day *L. maxima* cell suspensions, the optimal outcomes were achieved under both white and yellow LEDs. When evaluating nitrogen sources, it was observed that both KNO_3_ and NaNO_3_ resulted in similar performance, with no significant differences between them (Figure 1; Supplementary Table S1).

Specifically, the 27-day W-WN condition demonstrated a 40% and 45% decrease in OD when compared with W-NaNO_3_ and W-KNO_3_, respectively. Similarly, for the 27-day Y-WN condition, the OD showed a reduction of 34.4% and 38% when compared with Y-NaNO_3_ and Y-KNO_3_, respectively.

The growth rate trends like those observed for the optical density (OD), highlighting the superiority of white and yellow LEDs. Notably, over the 27-day period, the W-WN condition demonstrated a significant 12.6% increase in dry weight (DW) in comparison to W-NaNO_3_ (Figure 2, Supplementary Table S2). Remarkably, this improvement was not significantly different when compared to the KNO_3_ condition. Similarly, the Y-WN condition exhibited a noteworthy non-significant growth of 10.8% compared to Y-NaNO_3_. These results underscore the influential role of both light spectrum and nitrogen source on the growth dynamics *of Limnospira maxima*. The OD and dry weight (DW) exhibited moderate correlation at both the 12- and 27-day time points, revealing their interdependence as growth indicators. However, an intriguing observation emerged when all datasets were aggregated as input data: the correlation between these two variables intensified significantly (*r* = 0.798; *p* = 5.82 × 10^−41^) (Supplementary Data File). This suggests that the relationship between OD and DW is multifaceted, influenced not only by the growth period but also by various intrinsic and extrinsic factors. This enhanced correlation highlights the complexity of the interplay between growth kinetics and biomass accumulation in *L. maxima* under varying conditions.

### 3.3. Chlorophyll “a” (Chla) and Total Carotenoids (Car)

**Figure 3:**
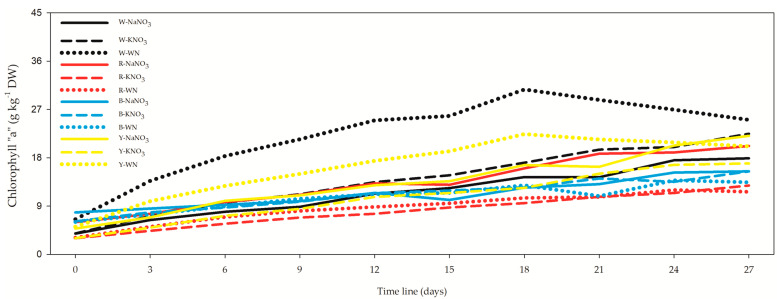
Influence of light spectra and nitrogen source on chlorophyll “a” concentration in *Limnospira maxima*. This figure illustrates the influence of varying light spectra and nitrogen sources on chlorophyll “a” concentration in *Limnospira maxima* cultures. The cultures were supplemented with NaNO_3_, KNO_3_, or maintained as controls (WN) under white, red, blue, and yellow light spectra. When specifically considering the 27-day *L. maxima* cell suspension, the most favorable outcomes were observed in the W-WN condition, followed by W-KNO_3_, Y-NaNO_3_, Y-WN, and R-NaNO_3_. Interestingly, blue LEDs and all other nitrogen sources under the red light consistently exhibited lower values throughout the study.

Chlorophyll “a” concentration showed variation, ranging from 2.99 ± 0.11 g kg^−1^ DW on day 0 in the R-KNO_3_ *L. maxima* cell suspension (Figure 3, Supplementary Table S3) to 30.70 ± 0.11 g kg^−1^ DW in a W-WN *L. maxima* cell suspension on day 18. Specifically considering the 27-day *L. maxima* cell suspension, W-WN exhibited the most favorable outcomes, followed by W-KNO_3_, Y-NaNO_3_, Y-WN, and R-NaNO_3_. Interestingly, blue LEDs and all other red LED nitrogen sources consistently displayed lower values throughout the study.

**Figure 4:**
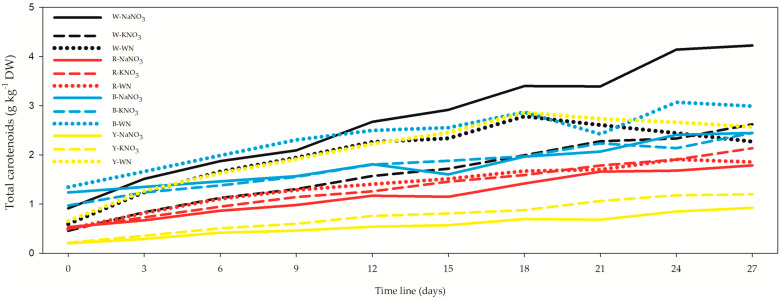
Effect of light spectra and nitrogen source on total carotenoid concentration in *Limnospira maxima.* This figure illustrates the influence of light spectra and nitrogen sources on the total carotenoid concentration in *Limnospira maxima* when grown under white, red, blue, and yellow light spectra. The cultures were supplemented with NaNO_3_, KNO_3_, and a control (WN). When specifically focusing on the 27-day cell suspensions, the highest carotenoid concentration was observed in the following order: W-NaNO_3_, B-WN, W-KNO_3_, and Y-WN.

Total carotenoids exhibited a notably distinct pattern compared to the previous results. The variation in carotenoid concentration spanned from 0.20 ± 0.01 g kg^−1^ DW in the Y-NaNO_3_ *L. maxima* cell suspension on day 0 (Figure 4, Supplementary Table S4) to 4.22 ± 0.101 g kg^−1^ DW in a W-NaNO_3_ *L. maxima* cell suspension on day 27. Specifically focusing on the 27-day cell suspensions, the order of highest Carotenoid concentration was observed as follows: W-NaNO_3_, B-WN, W-KNO_3_, and Y-WN.

The correlation between chlorophyll “a” (Chla) and optical density (OD) exhibited varying strengths over different time intervals. After 3 days, the correlation was non-significant (*r* = 0.312), and it remained weak after both 12 days (*r* = 0.312) and 27 days (*r* = 0.312). However, when considering the entire dataset with mean values, a more apparent correlation emerged (*r* = 0.669). Notably, the correlation between Chla and wet weight was consistently positive, with a moderately high strength after 27 days (*r* = 0.708), high after 3 days (*r* = 0.881) and 12 days (*r* = 0.878), and even stronger when all data were aggregated (*r* = 0.921).

The relationship between total carotenoids (Car) and OD or DW showed distinct patterns. For Car × OD after 3 and 12 days, the correlation was negative, while Car × DW displayed a positive correlation during these time frames. After 27 days, the correlation between Car × OD or Car × DW was not statistically significant. However, when aggregating all data, the correlation for Car × OD was weak (*r* = −0.252), and Car × DW exhibited a moderate correlation (*r* = 0.587). For additional details, refer to the Supplementary Data File.

### 3.4. Phycocyanin, Allophycocyanin, Phycoerythrin, and Soluble Proteins

**Figure 5:**
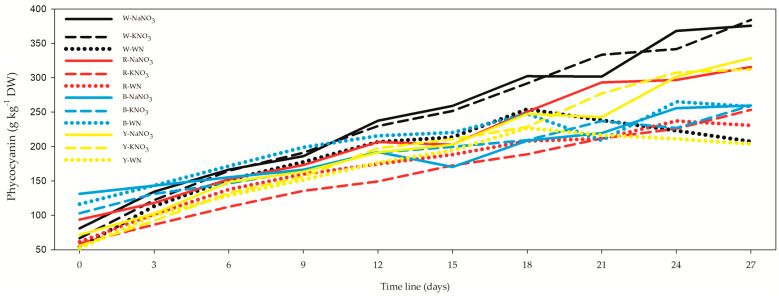
Impact of light spectra and nitrogen source on phycocyanin concentration in *Limnospira maxima* cultivation. This figure demonstrates the influence of various light spectra and nitrogen sources on the concentration of phycocyanin in *Limnospira maxima* cultures. The cultures were supplemented with NaNO_3_, KNO_3_, or maintained as controls (WN) under white, red, blue, and yellow light spectra. At the conclusion of the 27-day cultivation period, the sequence of phycocyanin concentration was as follows: W-KNO_3_, W-NaNO_3_, Y-NaNO_3_, R-NaNO_3_, Y-KNO_3_.

The concentration of phycocyanin exhibited a range from 52.02 ± 1.64 g kg^−1^ DW in the Y-WN *L. maxima* cell suspension at the outset (Figure 5, Supplementary Table S5) to 384.11 ± 9.52 g kg^−1^ DW in the W-KNO_3_ *L. maxima* cell suspension at the 27-day mark. At the conclusion of the 27-day cultivation period, the sequence of phycocyanin concentration was as follows: W-KNO_3_, W-NaNO_3_, Y-NaNO_3_, R-NaNO_3_, Y-KNO_3_. Notably, the W-WN treatment on day 27 demonstrated a reduction of 44.8% and 46% compared to W-NaNO_3_ and W-KNO_3_, respectively. Similarly, under red light on day 27, the R-WN treatment showed decreases of 26.9% (R-NaNO_3_) and 9% (R-KNO_3_). Conversely, the reduction seen in B-WN under blue light on day 27 was non-significant, with values dropping by only 0.6% (B-NaNO_3_) and 1.7% (B-KNO_3_). The Y-WN treatment under yellow light on day 27 displayed a decline to 38% and 34.8% of the values observed in Y-NaNO_3_ and Y-KNO_3_, respectively.

Regarding the correlation between phycocyanin and OD, it was weak and negative (*r* = −0.267), weak and positive (*r* = 0.252), moderate and positive (*r* = 0.663), and strong and negative (*r* = −0.825) for the 3-day, 12-day, and 27-day datasets and their combined data, respectively (Supplementary Data File).

**Figure 6:**
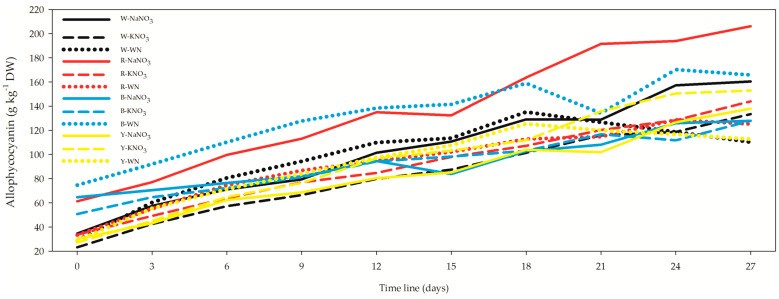
Effect of light spectra and nitrogen source on allophycocyanin concentration in *Limnospira maxima.* This figure examines the impact of different light spectra and nitrogen sources on the concentration of allophycocyanin in *Limnospira maxima* cultures. The cultures were supplemented with NaNO_3_, KNO_3_, or maintained as controls (WN) under white, red, blue, and yellow light spectra. Focusing exclusively on the 27-day treatments of *L. maxima* cell suspensions, it becomes evident that R-NaNO_3_ was the most productive treatment for allophycocyanin.

Allophycocyanin demonstrated a range from 23.13 ± 1.64 g kg^−1^ DW in the W-KNO_3_ *L. maxima* cell suspension at the commencement (Figure 6, Supplementary Table S6) to 206.12 ± 46.14 g kg^−1^ DW in the R-NaNO_3_ *L. maxima* cell suspension at the 27-day interval. Focusing solely on the 27-day *L. maxima* cell suspension treatments, it becomes evident that R-NaNO_3_ was the most productive treatment for allophycocyanin. Notably, the R-NaNO_3_ medium exhibited increments of 24.4%, 28.5%, 34.8%, and 43.3% compared to B-WN, W-NaNO_3_, Y-KNO_3_, and R-KNO_3_, respectively.

As for the correlation between allophycocyanin and OD, it displayed a moderately negative correlation (*r* = −0.633), indicating an inverse relationship.

**Figure 7:**
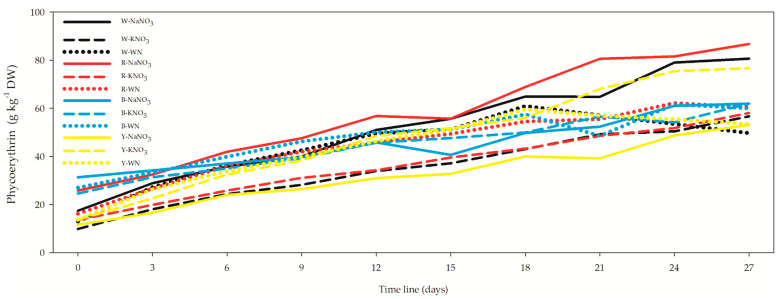
Effect of light spectra and nitrogen source on phycoerythrin concentration in *Limnospira maxima*. This figure explores the influence of different light spectra and nitrogen sources on the concentration of phycoerythrin in *Limnospira maxima* cultures. The cultures were supplemented with NaNO_3_, KNO_3_, or maintained as controls (WN) under white, red, blue, and yellow light spectra. Similar to allophycocyanin, phycoerythrin exhibited higher concentrations under the red LEDs.

The correlation between allophycocyanin and OD was moderately negative (*r* = −0.321) and non-significant (*p* = 0.834) for 3, 12, and 27 days. However, the correlation between allophycocyanin and OD became moderately strong (*r* = −0.709) when all data were combined. Phycoerythrin, as the third phycobiliprotein in descending order, exhibited a range from 9.81 ± 0.14 g kg^−1^ DW for W-KNO_3_ *L. maxima* cell suspension after 0 days (Figure 7, Supplementary Table S7) to 86.68 ± 2.20 g kg^−1^ DW for R-NaNO_3_ *L. maxima* cell suspension after 27 days. Similarly, to allophycocyanin, phycoerythrin showed higher concentrations under the red LEDs. In the *L. maxima* cell suspension after 27 days, R-NaNO_3_ provided 7.5%, 13.1%, and 40% higher phycoerythrin concentrations compared to W-NaNO_3_, Y-KNO_3_, and B-NaNO_3_, respectively. The correlation between phycoerythrin and OD was moderate and negative (*r* = −0.539), negative and weak (*r* = −0.186), and positive and non-significant (*p* = 0.164) for the 3-, 12-, and 27-day *L. maxima* cell suspensions. However, a strong and negative correlation (*r* = −0.654) between phycoerythrin and OD was observed when all datasets were combined.

**Figure 8:**
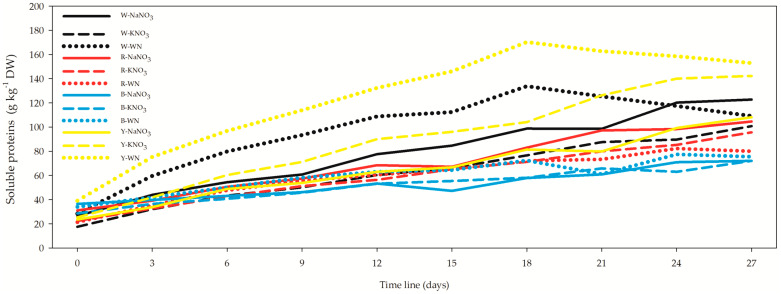
Effect of light spectra and nitrogen source on soluble protein concentration in *Limnospira maxima*. This figure investigates the influence of different light spectra and nitrogen sources on the concentration of soluble proteins in *Limnospira maxima* cultures. The cultures were supplemented with NaNO_3_, KNO_3_, or maintained as controls (WN) under white, red, blue, and yellow light spectra. Soluble proteins exhibited a distinct pattern when compared to the other proteins described earlier. In the case of the 27-day *L. maxima* cell suspension, the highest concentration was observed in the Y-WN condition.

Moreover, the results for soluble proteins displayed a completely different pattern compared to the other proteins described above (Figure 8, Supplementary Table S8). For an *L. maxima* cell suspension after 27 days, a higher value was obtained for Y-WN (152.86 ± 2.88 g kg^−1^ DW), which was 7.5%, 24.6%, 40.1%, and 41.5% higher than Y-KNO_3_, W-NaNO_3_, W-WN, and Y-NaNO_3_, respectively. The correlation between protein concentration and OD was moderate in the 12- (*r* = 0.515) and 27-day *L. maxima* cell suspension (*r* = 0.466), and strong in the 3-day suspension (*r* = 0.939), with *r* = 0.752 when all datasets were combined.

### 3.5. Principal Component Analysis (PCA)

**Figure 9:**
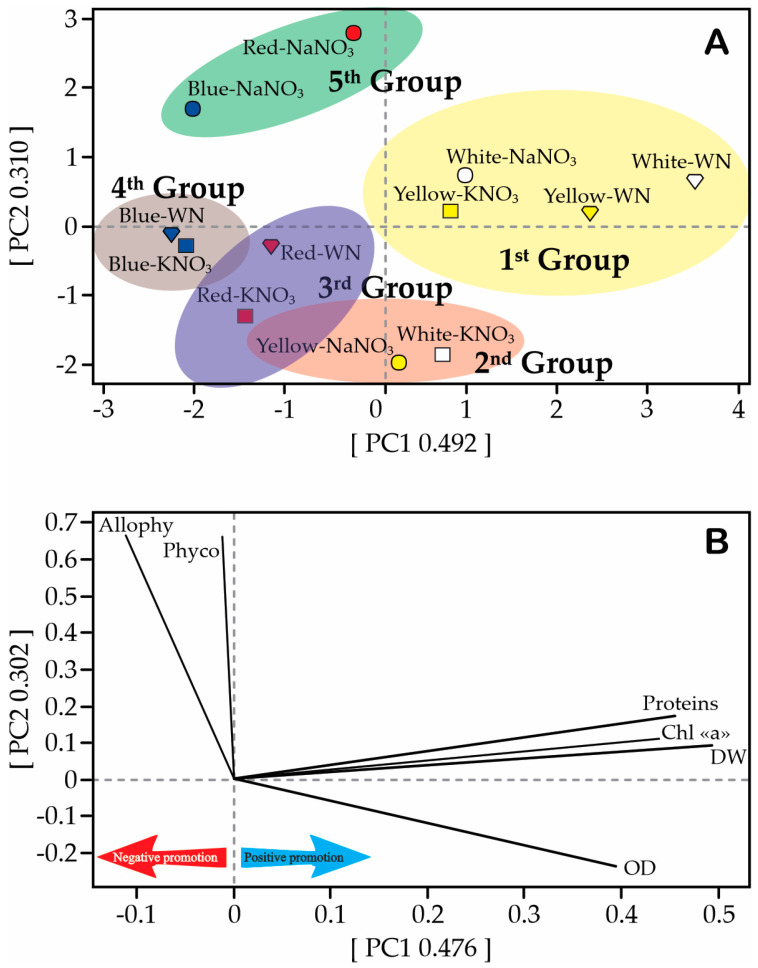
Multivariate analysis of light and nitrogen source effects on various features of *Limnospira maxima* cultivated in different light spectra (white, red, blue, and yellow), supplemented with NaNO_3_, KNO_3_, and control (WN). (**A**) The figure depicts a five-group clustering of treatments based on their similarities. (**B**) The diagram illustrates the strength of each feature’s influence on *L. maxima* growth, as observed through the increase in optical density (OD) and dry weight (DW). All measurements were taken after 27 days of *L. maxima* growth. Allophy refers to allophycocyanin; Phyco represents phycoerythrin; Proteins indicates soluble proteins; Chl “a” signifies chlorophyll a.

The principal component analysis (PCA) reveals the formation of data clusters into five distinct groups encompassing all treatments. The first group amalgamates four treatments with shared characteristics (W-NaNO_3_, W-WN, Y-KNO_3_, and Y-WN), while the second group aligns W-KNO_3_ and Y-NaNO_3_. The third group aggregates R-KNO_3_ and R-WN, the fourth group aligns B-KNO_3_ and B-WN, and the fifth group merges R-NaNO_3_ and B-NaNO_3_ (Figure 9). Figure 9B indicates that the vectors corresponding to OD, DW and Chla significantly contribute to the formation of the first group. Conversely, the presence of allophycocyanin and phycoerythrin chiefly influences the formation of the fifth group. Notably, groups 2, 3, and 4 lack distinct defining characteristics. These three groups exhibit substantial interconnectedness, with their dendrogram reflecting a lack of pronounced similarities among all indices above a threshold of 59.2 (Supplementary Table S3).

The Pearson correlation analysis reveals strong correlations (*r* ≥ 0.850) among all factors. Notably, optical density (OD) exhibits a negative correlation with total carotenoids, phycocyanin, allophycocyanin, and phycoerythrin (Supplementary Data File). Therefore, it is reasonable to anticipate that changes in one characteristic would correspond to changes in others. This anticipation aligns with the actual observations, leading us to determine that the treatment involving yellow or white light supplemented with KNO_3_ is the most favorable. It is suggested that even if the outcomes deviate from initial expectations, this treatment remains the optimal choice (further elaborated in the discussion section).

## 4. Discussion

The cyanobacteria *L. maxima* has evolved unique physiological and morphological characteristics that allow them to respond to changes in environmental parameters, such as nutrients and light [23]. In our study, we contribute novel findings that shed light on the intricate interactions between light spectra, nitrogen sources, and various physiological features of *L. maxima*. Our results reveal that when comparing *L. maxima* cell suspensions after 27 days, white and yellow LEDS promotes the most favorable OD results. Moreover, the choice of nitrogen source, either KNO_3_ or NaNO_3_, yielded growth outcomes without significant differences. The observation of higher growth (higher OD) and increased chlorophyll content under white light corroborates findings by Madhyastha and Vatsala [24] on pigment production in *Spirulina fussiformis*, where various conditions were explored. Similarly, our results are aligned with those of Ortiz-Moreno et al. [25], who studied *Nostoc ellipsosporum*’s growth in relation to light wavelengths and concluded that white light followed by yellow light resulted in optimal cell growth.

In contrast, blue light showed a marked inhibitory effect on the growth of *N. ellipsosporum*. Khatoon et al. [26] determined the effect of different light sources and media (wastewater and Bold’s Basal Medium—BBM) on the growth and production of phyco-biliproteins in *Pseudanabaena mucicola*, describing that white light had a significantly higher growth rate in terms of OD compared to blue light and natural light. The highest dry biomass was also produced when *P. mucicola* was grown under a white light source, with no significant difference between BBM and wastewater media.

Contrary to our results, some studies have reported red light as promoting the highest growth, while blue light exhibited inhibitory effects [14,15,27,28,29]. The disparity in out-comes might be attributed to variations in light sources, cyanobacterial species (*A. platensis*, *L. maxima*, *A. fusiformis*), culture medium composition, pH levels, aeration rates, and other factors [27]. Our results also differ significantly from the Bahman et al. [30], who describes a higher growth rate of *Arthrospira platensis* under blue light.

It should be noted that nitrogen plays a pivotal role in biomass and pigment production in *L. maxima*. Additionally, nitrogen usage holds economic significance in large-scale cultivation [13,31]. Our study demonstrates that after 27 days, both KNO_3_ and NaNO_3_ yield similar growth, as indicated by optical density measurements. Notably, KNO_3_, being more cost-effective, produces comparable cell growth to NaNO_3_, which is comparatively more expensive. These findings are in line with those of Mousavi et al. [13], who studied the effect of various nitrogen sources on phycocyanin production in *A. platensis* and found that NaNO_3_- and KNO_3_-supplemented media resulted in the highest biomass production.

An increase in dry weight (DW) production under white and yellow lights, followed by a subsequent decrease, was observed in our study. This behavior was reported by Pelagatti et al. [32] in their research of the effects of yellow and blue light on the biochemical characteristics of *Limnospira fusiformis*. Notably, exposure to yellow light led to higher biomass production compared to blue light (Table 2). Similar findings were documented by DeMooij et al. [33], who explored *Chlamydomonas reinhardtii’s* productivity concerning biomass-specific light absorption rates. They found that yellow light yielded the highest productivity per area, followed by white light, indicating its efficiency in biomass production.

Regarding nitrogen functionality, Möllers et al. [34] noted that nitrogen limitation in *Synechococcus* sp. enhanced the carbon–nitrogen ratio and glycogen content, thus improving its viability as a biomass feedstock. Similarly, Cuellar-Bermudez et al. [35] found that nitrogen-poor conditions prompted Arthrospira to accumulate carbohydrates while reducing protein content. This adaptation to nitrogen starvation results in metabolic shifts, amino acid balance, and carbon metabolism reconfiguration [36]. Our findings align with these insights, as we observed similar responses in *L. maxima* under varying nitrogen conditions.

For the functionality of nitrogen, Möllers et al. [34] found that the nitrogen limitation on *Synechococcus* sp. was used to increase the carbon–nitrogen ratio and glycogen content to improve its utility as a biomass feedstock. In corroboration, Cuellar-Bermudez et al. [35], found that when *Arthrospira* is grown under nitrogen-poor conditions, it reduces the protein content and accumulates carbohydrates (up to 57–77% of dry weight), generally glycogen [37]. Similar results have been shared by Saxena et al. [38] in *A. platensis*. Acclimatization to N starvation is probably the best example of complexity in molecular responses. The biological response varies from metabolic changes to cell differentiation. During N starvation, the central metabolism is often reset, including energy production and energy conversion, amino acid metabolism, carbohydrate transport, and other types of metabolism. In fact, two simultaneous phenomena have been described: (i) the optimization and use of intracellular nitrogenous components (e.g., cyanophycin and phycobilisomes) and (ii) the redesign of the carbon metabolism to deal with excess carbon [36]. Also, N deficiencies induce phycobilisome degradation that is distinguished by a color change of the culture from blue-green to a paler shade [39]. In addition, some studies have shown that when limited by extracellular nitrogen, cyanophycin granules are degraded to meet the demands of metabolic nitrogen [40]. In addition, a proteomic analysis has shown that an abundance of cyanophykinase is greater under N-stressed conditions, providing a large amount of arginine and aspartate that are more likely to balance against an intracellular N deficiency [41].

Chlorophyll “a” concentration in our study displayed a pattern similar to DW. Higher concentrations were noted on the 18th day under white and yellow light in the absence of nitrogen. Comparable observations were made by Madhyastha and Vatsala [24], Milia et al. [28], and others, demonstrating a preference for white and orange lights in promoting chlorophyll “a” level (Table 2).

**Table 2:** Physiological parameters of cyanobacterial species and optimal growth conditions.

Species	OD	Dry Weight	Chl a	Carotenoids	Phycocyanin	Allophycocyanin	Phycoerythrin	Soluble Proteins	Source	Conditions That Yielded the Highest Values
*Limnospira maxima*	1.31	1.76 g L^−1^	30.70 g kg^−1^	4.22 g kg^−1^	384.11 g k g^−1^	206.12 g kg^−1^	86.68 g kg^−1^	152.86 g kg^−1^	This research.	W-KNO_3_ and Y-KNO_3_: OD; W-WN: Dry weight and Chla; W-NaNO_3_: Carotenoids; W-KNO_3_: phycocyanin; R-NaNO_3_: allophycocyanin and phycoerythrin; Y-WN: proteins.
*Arthrospira maxima*	-	-	0.51 g kg^−1^	-	3.20 g kg^−1^	0.19 g kg^−1^	0.97 g kg^−1^	-	Park and Dinh [15]	Red and green light: Chl a; blue light: phycobiliproteins. Nitrogen source: NaNO_3_
*Spirulina fussiformis*	-	0.8 g L^−1^	0.0055 g L^−1^	-	2.5 g L^−1^	-	-	-	Madhyastha and Vatsala [24]	White light: Dry weight and Chla; green light: phycocyanin; Nitrogen source: NaNO_3_
*Arthrospira maxima*	-	3.8133 g L^−1^	0.065 g L^−1^	0.01 g L^−1^	140 g kg^−1^	30 g kg^−1^	-	735.3 g kg^−1^	Milia et al. [28]	Orange light: Dry weight; White and orange lights: Chla; Blue light: proteins, phycocyanin and allophycocyanin. Nitrogen source: NaNO_3_
*Pseudanabaena mucicola*	0.731	0.55 g L^−1^	0.0021 g m^−2^	-	0.4 g L^−1^	0.523 g L^−1^	0.05 g L^−1^	-	Khatoon et al. [26]	White light and medium supplemented with NaNO_3_: OD; White light using wastewater: Dry weight, Chla, allophycocyanin and phycoerythrin; Blue light and medium supplemented with NaNO_3_: phycocyanin.
*Limnospira fusiformis*		0.3789 g L^−1^	0.00314 g L^−1^	0.00151 g L^−1^	0.01216 g L^−1^	-	-	0.1507 g L^−1^	Pelagatti et al. [32]	Yellow light: Chla, Dry weight, proteins and carotenoids; Blue light: phycocyanin. Nitrogen source: NaNO_3_.
*Arthrospira platensis*	1.706	1.415 g L^−1^	25.31 g kg^−1^	6.38 g kg^−1^	167.07 g kg^−1^	-	-	-	Lima et al. [42]	Lights composed of 70% red and 30% blue (R70:B30): OD, dry weight, Chla and carotenoids; Light deep red (DR): phycocyanin. Nitrogen source: KNO_3_.

Phycocyanin levels in our study peaked under white light, followed by yellow light with NaNO_3_ or KNO_3_ supplementation. Our findings highlight the potential of KNO_3_ as a cost-effective alternative to NaNO_3_ in promoting significant phycocyanin production. Interestingly, phycocyanin production responses to light color are varied in the literature [36,39,43,44], emphasizing the complexity of these interactions. The best results for white light on phycocyanin production were also reported in the study by Ojit et al. [45], who describe a higher concentration of this pigment after 15 days of culture in the cyanobacterium *Anabaena circinalis*. In contrast, they found the least amount of phycocyanin under blue light. Similarly, Park and Dinh [15] described a higher production of phycocyanin in L. maxima in cultures exposed to blue light. Likewise, Milia et al. [28] reported greater production of this pigment under blue light.

Some scholars have shown that blue light positively influences phycocyanin production [36,39,43,44]. In contrast, white light covers a broader range of wavelengths and does not specifically increase phycocyanin production. Furthermore, the phycocyanin-to-chlorophyll a ratio and the allophycocyanin-to-chlorophyll a ratio were higher under blue light than under orange or white light for all strains, indicating that phycobilin content in-creased relative to Chl a when cells were exposed to blue light. Blue light stimulates *L. maxima* to produce more phycobilins to compensate for the lack of energy related to the limited range of radiation (400–475 nm), resulting in increased protein production [28]. In our study, blue light was overwhelmed by white, yellow, and sometimes red light.

In a wide range of literature reviews, *L. maxima* exhibits a remarkable ability to withstand nitrogen starvation, enabling it to thrive in various environments. Despite facing nutrient limitations, *L. maxima* displays exceptional adaptability and continues its growth. Regarding the absence of nitrogen, the cultures exposed to white and yellow light in the absence of nitrogen showed a decrease in the concentration of phycocyanin from the 18th day, while the treatments exposed to red light began to decrease after the 24th day. Joseph et al. [46] describe that during prolonged nitrogen deprivation, the cyanobacterium *Synechocystis* sp. PCC 6803 stores glycogen and degrades nitrogen-rich phycobilisomes, resulting in a loss of the pigment phycocyanin, a condition known as photo-blanching or chlorosis. Phycobilisome proteins constitute about 50% of the total protein in the cyanobacterial cell under optimal growth conditions and therefore can provide massive amounts of nutrients along with a degradation in response to N starvation [21]. In the absence of N, protein synthesis is limited, and photosynthetic energy and carbon are diverted from the synthesis of protein to carbohydrate production [35]. However, *L. maxima* actively degrades its proteins and recycles the nitrogen associated with amino acids. This indicates that *L. maxima* could degrade its phycobilisomes to supply nitrogen for other metabolic processes. The degradation of the phycobilisome could be in accordance with the observed reduction in phycocyanin content.

Cyanobacteria, particularly spirulina, harbor substantial economic potential as a sustainable source of valuable bioproducts, including proteins and phycocyanins. The market for these proteins is projected to reach USD 840 million in 2023, while phycocyanin is expected to reach USD 409.8 million in 2030 [47]. Cultivation systems, whether open or closed, come with distinct advantages and disadvantages. Hybrid systems, such as two-stage cultivation, hold promise for optimizing metabolite accumulation and biomass productivity. Spirulina-based biorefining is a growing trend, capable of producing high-value proteins and phycocyanins. However, life cycle assessments and techno-economic analyses are essential to ensure viability. The implementation of Spirulina-based biorefineries in a circular bioeconomy is on the rise, supported by research indicating economic feasibility through the reduction of current operating costs, utilization of low-cost inputs, and minimization of non-automated work for upstream processes [11].

Finally, it is recommended to conduct gene expression studies in *L. maxima* to identify and characterize the genes involved in the synthesis of phycocyanin and other metabolites of commercial significance. This will help understand the regulatory mechanisms and factors influencing the production of these valuable compounds. Additionally, an analysis of the enzymatic and non-enzymatic defense system in *L. maxima*, particularly in response to light and nitrogen availability stress, is suggested. This would encompass the evaluation of antioxidant enzymes, antioxidant metabolites, and other key components of the defense system to comprehend how the cyanobacteria adapts and responds to environmental conditions. Furthermore, investigating specific genes involved in the expression of the defense system in *L. maxima* in response to light and nitrogen sources is crucial. This would involve identifying and characterizing transcriptional regulators and response elements within the cyanobacteria’s genome, providing a more comprehensive insight into the underlying molecular mechanisms of stress response.

## 5. Conclusions

In this study, we delved into the intricate interplay between environmental factors, particularly light and nitrogen availability, and the physiological responses of *Limnospira maxima*. The presented data underscores the significance of white and yellow light in promoting substantial growth and chlorophyll content, aligning with previous studies in the field. A comparison of nitrogen sources reveals that both KNO_3_ and NaNO_3_ exhibit similar effects on cell growth, with KNO_3_ emerging as the more cost-effective option for large-scale cultivation. Our investigation into pigment production highlights the prominence of white light in enhancing phycocyanin levels, a critical pigment with implications for both nutritional and industrial applications. However, the nuanced influence of light color on phycocyanin content requires further exploration, given divergent results in the literature.

The remarkable adaptability of *L. maxima* to varying nitrogen conditions serves as a testament to its resilience and intricate metabolic responses. The discerned patterns of pigment degradation in the absence of nitrogen shed light on the organism’s dynamic nature, where phycobilisomes are strategically harnessed as repositories of both nitrogen and energy. These insights deepen our comprehension of *L. maxima’s* adept survival strategies and open new avenues for probing its potential role in nutrient recycling and adaptive metabolic responses.

In conclusion, our study uncovers the intricate relationships between light, nitrogen, and physiological responses in *Limnospira maxima.* These findings have implications for sustainable food production and resource management, particularly in the face of climate change and resource limitations. Through unraveling the adaptive strategies of cyanobacteria like *L. maxima*, we contribute to the broader discourse on sustainability and offer insights into potential solutions for addressing global food security challenges. The study of cyanobacteria’s metabolic flexibility and responses provides a promising avenue for revolutionizing food production and resource utilization in the pursuit of a more sustainable future.

## Figures and Tables

**Table 1:** Characteristics of light sources and measured photosynthetic active radiation (PAR).

Light Source	Model	Trademark	Color	Power	PAR (μmol m^−2^ s^−1^)	
					Theoretical	Measured ^1^
LED T5	M22	Mercury, Kwangchow, China	Write—W	18 W	82.8	79.14 ± 1.94
LED T5	M29	Mercury, Kwangchow, China	Red—R	18 W	82.8	77.51 ± 2.23
LED T5	M21	Mercury, Kwangchow, China	Blue—B	18 W	82.8	78.10 ± 1.81
LED T5	M24	Mercury, Kwangchow, China	Yellow—Y	18 W	82.8	79.31 ± 1.85

^1^ All value denotes mean (±SE), *n* = 15, *p* value = 0.162 (ns).

## Data Availability

Data are contained within the article and supplementary materials.

## Supplementary Materials

The following supporting information can be downloaded at: https://www.mdpi.com/article/10.3390/biology12121462/s1, Supplementary Figure S1: Experimental design; Supplementary Table S1. Effect of light spectra and nitrogen source on optical density of *Limnospira maxima* cultures were grown under white, red, blue, and yellow light spectra, supplemented with NaNO_3_, KNO_3_, and a control (WN). The values presented in the figure indicate the means (±standard error). Capital letters indicate significant effects between nitrogen sources within the same light spectrum, while lowercase letters indicate significance between light spectra within the same nitrogen source. The statistical significance was determined using the SNK test. Supplementary Table S2. Effect of light spectra and nitrogen source on dry weight of *Limnospira maxima* cultures were grown under white, red, blue, and yellow light spectra, supplemented with NaNO_3_, KNO_3_, and a control (WN). The values presented in the figure indicate the means (±standard error). Capital letters indicate significant effects between nitrogen sources within the same light spectrum, while lowercase letters indicate significance between light spectra within the same nitrogen source. The statistical significance was determined using the SNK test. Supplementary Table S3. Effect of light spectra and nitrogen source on chlorophyll “a” concentration of *Limnospira maxima* cultures were grown under white, red, blue, and yellow light spectra, supplemented with NaNO_3_, KNO_3_, and a control (WN). The values presented in the figure indicate the means (±standard error). Capital letters indicate significant effects between nitrogen sources within the same light spectrum, while lowercase letters indicate significance between light spectra within the same nitrogen source. The statistical significance was determined using the SNK test. Supplementary Table S4. Effect of light spectra and nitrogen source on total carotenoids concentration of *Limnospira maxima* grown in white, red, blue, and yellow light spectra, supplemented with NaNO_3_, KNO_3_, plus control (WN). Values followed by capital letter denote significative effect between N source in the same light spectra and small letter denote significance between light spectra in the same nitrogen source. All value represents media (±SD), followed by SNK statistical test. Supplementary Table S5. Impact of Light Spectra and Nitrogen Source on Phycocyanin Concentration in *Limnospira maxima* Cultivation. This figure showcases the influence of different light spectra and nitrogen sources on the concentration of phycocyanin in *Limnospira maxima* cultures. The cultures were supplemented with NaNO_3_, KNO_3_, or maintained as controls (WN) under white, red, blue, and yellow light spectra. The presented values denote the means along with their respective standard deviations (±SD). Capital letters indicate significant effects among nitrogen sources within the same light spectra, while lowercase letters denote significant differences among light spectra within the same nitrogen source. The statistical significance was determined through the SNK test. Supplementary Table S6. Effect of light spectra and nitrogen source on allophycocyanin concentration of *Limnospira maxima* grown in white, red, blue, and yellow light spectra, supplemented with NaNO_3_, KNO_3_, plus control (WN). Values followed by capital letters denote significant effects between N sources in the same light spectra, and small letters denote significance between light spectra in the same nitrogen source. All values represent means (±SD), followed by SNK statistical test. Supplementary Table S7. Effect of light spectra and nitrogen source on phycoerytrin concentration of *Limnospira maxima* grown in white, red, blue, and yellow light spectra, supplemented with NaNO_3_, KNO_3_, plus control (WN). Values followed by capital letter denote significative effect between N source in the same light spectra and small letter denote significance between light spectra in the same nitrogen source. All value represents media (±SD), followed by SNK statistical test. Supplementary Table S8. Effect of light spectra and nitrogen source on soluble proteins concentration of *Limnospira maxima* grown in white, red, blue, and yellow light spectra, supplemented with NaNO_3_, KNO_3_, plus control (WN). Values followed by a capital letter denote a significant effect between N source in the same light spectra, and a small letter denotes significance between light spectra in the same nitrogen source. All values represent the mean (±SD), followed by the SNK statistical test.

## References

[1] Azadi H., Ghazali S., Ghorbani M., Tan R., Witlox F. (2023). Contribution of Small-scale Farmers to Global Food Security: A Meta-analysis. J. Sci. Food Agric..

[2] FAO, FIDA, OMS, PMA, UNICEF (2022). El Estado de la Seguridad Alimentaria y la Nutrición en el Mundo 2022.

[3] Ravindran B., Gupta S., Cho W.-M., Kim J., Lee S., Jeong K.-H., Lee D., Choi H.-C. (2016). Microalgae Potential and Multiple Roles—Current Progress and Future Prospects—An Overview. Sustainability.

[4] Yin C., Daoust K., Young A., Tebbs E., Harper D. (2017). Tackling Community Undernutrition at Lake Bogoria, Kenya: The Potential of Spirulina (*Arthrospira Fusiformis*) as a Food Supplement. Afr. J. Food Agric. Nutr. Dev..

[5] Soni R.A., Sudhakar K., Rana R.S. (2017). Spirulina—From Growth to Nutritional Product: A Review. Trends Food Sci. Technol..

[6] García-López D.A., Olguín E.J., González-Portela R.E., Sánchez-Galván G., De Philippis R., Lovitt R.W., Llewellyn C.A., Fuentes-Grünewald C., Parra Saldívar R. (2020). A Novel Two-Phase Bioprocess for the Production of *Arthrospira* (Spirulina) *maxima* LJGR1 at Pilot Plant Scale during Different Seasons and for Phycocyanin Induction under Controlled Conditions. Bioresour. Technol..

[7] Dolganyuk V., Sukhikh S., Kalashnikova O., Ivanova S., Kashirskikh E., Prosekov A., Michaud P., Babich O. (2023). Food Proteins: Potential Resources. Sustainability.

[8] Kulshreshtha A., Zacharia A.J., Jarouliya U., Bhadauriya P., Prasad G., Bisen P. (2008). Spirulina in Health Care Management. Curr. Pharm. Biotechnol..

[9] Lafarga T., Mayre E., Echeverria G., Viñas I., Villaró S., Acién-Fernández F.G., Castellari M., Aguiló-Aguayo I. (2019). Potential of the Microalgae Nannochloropsis and Tetraselmis for Being Used as Innovative Ingredients in Baked Goods. LWT Food Sci. Technol..

[10] Gal J.L., Cole N.R., Eggett D.L., Johnson S.M. (2023). Growth Comparison of *Arthrospira Platensis* in Different Vessels: Standard Cylinder vs. Enhanced Surface Area at Low Light. Appl. Phycol..

[11] Vieira Costa J.A., Freitas B.C.B., Rosa G.M., Moraes L., Morais M.G., Mitchell B.G. (2019). Operational and Economic Aspects of Spirulina-Based Biorefinery. Bioresour. Technol..

[12] Wang J., Cheng W., Liu W., Wang H., Zhang D., Qiao Z., Jin G., Liu T. (2019). Field Study on Attached Cultivation of *Arthrospira* (Spirulina) with Carbon Dioxide as Carbon Source. Bioresour. Technol..

[13] Mousavi M., Mehrzad J., Najafi M.F., Zhiani R., Shamsian S.A.A. (2022). Nitrate and Ammonia: Two Key Nitrogen Sources for Biomass and Phycocyanin Production by *Arthrospira* (Spirulina) *platensis*. J. Appl. Phycol..

[14] da Fontoura Prates D., Radmann E.M., Duarte J.H., de Morais M.G., Costa J.A.V. (2018). Spirulina Cultivated under Different Light Emitting Diodes: Enhanced Cell Growth and Phycocyanin Production. Bioresour. Technol..

[15] Park J., Dinh T.B. (2019). Contrasting Effects of Monochromatic LED Lighting on Growth, Pigments and Photosynthesis in the Commercially Important Cyanobacterium *Arthrospira maxima*. Bioresour. Technol..

[16] Garcia A.K., McShea H., Kolaczkowski B., Kaçar B. (2020). Reconstructing the Evolutionary History of Nitrogenases: Evidence for Ancestral Molybdenum-cofactor Utilization. Geobiology.

[17] Bachchhav M.B., Kulkarni M.V., Ingale A.G. (2017). Enhanced Phycocyanin Production from *Spirulina platensis* Using Light Emitting Diode. J. Inst. Eng. India Ser. E.

[18] Pineda-Rodriguez Y.Y., Pompelli M.F., Jarma-Orozco A., Rodríguez N.V., Rodriguez-Paez L.A. (2023). A New and Profitable Protocol to DNA Extraction in *Limnospira maxima*. Methods Protoc..

[19] Rajasekaran C., Ajeesh C.P.M., Balaji S., Shalini M., SIVA R., Das R., Fulzele D.P., Kalaivani T. (2016). Effect of Modified Zarrouk’s Medium on Growth of Different Spirulina Strains. Walailak J. Sci. Technol. WJST.

[20] Wellburn A.R. (1994). The Spectral Determination of Chlorophylls a and b, as Well as Total Carotenoids, Using Various Solvents with Spectrophotometers of Different Resolution. J. Plant Physiol..

[21] Lichtenthaler H.K., Buschmann C. (2001). Chlorophylls and Carotenoids: Measurement and Characterization by UV-VIS Spectroscopy. Curr. Protoc. Food Anal. Chem..

[22] Bennett A., Bogorad L. (1973). Complementary Chromatic Adaptation in a Filamentous Blue-Green Alga. J. Cell Biol..

[23] Tandeau N., Houmard J. (1993). Adaptation of Cyanobacteria to Environmental Stimuli: New Steps towards Molecular Mechanisms. FEMS Microbiol. Lett..

[24] Madhyastha H.K., Vatsala T.M. (2007). Pigment Production in *Spirulina fussiformis* in Different Photophysical Conditions. Biomol. Eng..

[25] Ortiz-Moreno M.L., Cárdenas-Poblador J., Agredo J., Solarte-Murillo L.V. (2020). Modeling the Effects of Light Wavelength on the Growth of *Nostoc ellipsosporum*. Univ. Sci..

[26] Khatoon H., Kok Leong L., Abdu Rahman N., Mian S., Begum H., Banerjee S., Endut A. (2018). Effects of Different Light Source and Media on Growth and Production of Phycobiliprotein from Freshwater Cyanobacteria. Bioresour. Technol..

[27] Jung C.H.G., Waldeck P., Sykora S., Braune S., Petrick I., Küpper J.-H., Jung F. (2022). Influence of Different Light-Emitting Diode Colors on Growth and Phycobiliprotein Generation of *Arthrospira Platensis*. Life.

[28] Milia M., Corrias F., Addis P., Chini Zitelli G., Cicchi B., Torzillo G., Andreotti V., Angioni A. (2022). Influence of Different Light Sources on the Biochemical Composition of *Arthrospira* spp. Grown in Model Systems. Foods.

[29] Tayebati H., Pajoum Shariati F., Soltani N., Sepasi Tehrani H. (2021). Effect of Various Light Spectra on Amino Acids and Pigment Production of *Arthrospira Platensis* Using Flat-Plate Photobioreactor. Prep. Biochem. Biotechnol..

[30] Bahman M., Aghanoori M., Jalili H., Bozorg A., Danaee S., Bidhendi M.E., Amrane A. (2022). Effect of Light Intensity and Wavelength on Nitrogen and Phosphate Removal from Municipal Wastewater by Microalgae under Semi-Batch Cultivation. Environ. Technol..

[31] Mirhosseini N., Cano-Europa E., Davarnejad R., Hallajisani A., Franco-Colín M., Tavakoli O., Blas-Valdivia V. (2021). Cultivations of *Arthrospira maxima* (Spirulina) Using Ammonium Sulfate and Sodium Nitrate as an Alternative Nitrogen Sources. Iran. J. Fish. Sci..

[32] Pelagatti M., Mori G., Falsini S., Ballini R., Lazzara L., Papini A. (2023). Blue and Yellow Light Induce Changes in Biochemical Composition and Ultrastructure of *Limnospira fusiformis* (Cyanoprokaryota). Microorganisms.

[33] De Mooij T., De Vries G., Latsos C., Wijffels R.H., Janssen M. (2016). Impact of Light Color on Photobioreactor Productivity. Algal Res..

[34] Möllers K.B., Cannella D., Jørgensen H., Frigaard N.-U. (2014). Cyanobacterial Biomass as Carbohydrate and Nutrient Feedstock for Bioethanol Production by Yeast Fermentation. Biotechnol. Biofuels.

[35] Cuellar-Bermudez S.P., Struyf T., Versluys M., Van Den Ende W., Wattiez R., Muylaert K. (2022). Quantification of Extracellular and Biomass Carbohydrates by *Arthrospira* under Nitrogen Starvation at Lab-Scale. Algal Res..

[36] Deschoenmaeker F., Facchini R., Cabrera Pino J.C., Bayon-Vicente G., Sachdeva N., Flammang P., Wattiez R. (2016). Nitrogen Depletion in *Arthrospira* sp. PCC 8005, an Ultrastructural Point of View. J. Struct. Biol..

[37] Liu Q., Yao C., Sun Y., Chen W., Tan H., Cao X., Xue S., Yin H. (2019). Production and Structural Characterization of a New Type of Polysaccharide from Nitrogen-Limited *Arthrospira platensis* Cultivated in Outdoor Industrial-Scale Open Raceway Ponds. Biotechnol. Biofuels.

[38] Saxena R., Rodríguez-Jasso R.M., Chávez-Gonzalez M.L., Aguilar C.N., Quijano G., Ruiz H.A. (2022). Strategy Development for Microalgae *Spirulina platensis* Biomass Cultivation in a Bubble Photobioreactor to Promote High Carbohydrate Content. Fermentation.

[39] Sendersky E., Kozer N., Levi M., Moizik M., Garini Y., Shav-Tal Y., Schwarz R. (2015). The Proteolysis Adaptor, NblA, Is Essential for Degradation of the Core Pigment of the Cyanobacterial Light-harvesting Complex. Plant J..

[40] Deschoenmaeker F., Facchini R., Leroy B., Badri H., Zhang C.-C., Wattiez R. (2014). Proteomic and Cellular Views of *Arthrospira* Sp. PCC 8005 Adaptation to Nitrogen Depletion. Microbiology.

[41] Wegener K.M., Singh A.K., Jacobs J.M., Elvitigala T., Welsh E.A., Keren N., Gritsenko M.A., Ghosh B.K., Camp D.G., Smith R.D. (2010). Global Proteomics Reveal an Atypical Strategy for Carbon/Nitrogen Assimilation by a Cyanobacterium under Diverse Environmental Perturbations. Mol. Cell. Proteom..

[42] Lima G.M., Teixeira P.C.N., Teixeira C.M.L.L., Filócomo D., Lage C.L.S. (2018). Influence of Spectral Light Quality on the Pigment Concentrations and Biomass Productivity of *Arthrospira platensis*. Algal Res..

[43] Zittelli G., Mugnai G., Milia M., Cicchi B., Silva A.M., Angioni A., Addis P., Torzillo G. (2022). Effects of Blue, Orange and White Lights on Growth, Chlorophyll Fluorescence, and Phycocyanin Production of *Arthrospira platensis* Cultures. Algal Res..

[44] Lee S.-H., Lee J.E., Kim Y., Lee S.-Y. (2016). The Production of High Purity Phycocyanin by *Spirulina platensis* Using Light-Emitting Diodes Based Two-Stage Cultivation. Appl. Biochem. Biotechnol..

[45] Ojit S.K., Indrama T., Gunapati O., Avijeet S.O., Subhalaxmi S.A., Silvia C., Indira D.W., Romi K., Minerva S., Thadoi D.A. (2015). The Response of Phycobiliproteins to Light Qualities in *Anabaena Circinalis*. J. Appl. Biol. Biotechnol..

[46] Joseph A., Aikawa S., Sasaki K., Matsuda F., Hasunuma T., Kondo A. (2014). Increased Biomass Production and Glycogen Accumulation in apcE Gene Deleted *Synechocystis* Sp. PCC 6803. AMB Express.

[47] Thevarajah B., Nishshanka G.K.S.H., Premaratne M., Nimarshana P.H.V., Nagarajan D., Chang J.-S., Ariyadasa T.U. (2022). Large-Scale Production of Spirulina-Based Proteins and c-Phycocyanin: A Biorefinery Approach. Biochem. Eng. J..

