# Supplementary material for: Optimal Laboratory Cultivation Conditions of Limnospira maxima for Large-Scale Production
Source: Biology (Basel). 2023 Nov 24;12(12):1462. doi: 10.3390/biology12121462 (PMC10740766; doi:10.3390/biology12121462)
Supplement: Supplementary file 1 [file biology-12-01462-s001.zip › Supplementary Table S1.pdf]

Supplementary Table S1. Effect of light spectra and nitrogen source on optical density of *Limnospira maxima* cultures were grown under white, red, blue, and yellow light spectra, supplemented with $NaNO_3$, KNO_3, and a control (WN). The values presented in the figure indicate the means (± standard error). Capital letters indicate significant effects between nitrogen sources within the same light spectrum, while lowercase letters indicate significance between light spectra within the same nitrogen source. The statistical significance was determined using the SNK test.

Light spectra	N source	0	3	6	9	12	15	18	21	24	27
White	$NaNO_3$	0.26 Aa	0.43 Aa	0.53 Aa	0.59 Aa	0.76 Ba	0.83 Ba	0.97 Aa	0.96 Ba	1.18 Aa	1.20 Ba
	KNO_3	0.23 Ba	0.42 Aa	0.56 Aa	0.65 Aa	0.78 Aa	0.85 Aa	0.99 Aa	1.14 Aa	1.16 Ab	1.31 Aa
	WN	0.19 Ba	0.40 Aa	0.53 Aa	0.62 Aa	0.72 Ba	0.75 Bb	0.89 Bb	0.83 Cb	0.78 Ba	0.72 Cc
Red	$NaNO_3$	0.27 Aa	0.34 Ab	0.44 Ab	0.5 Ab	0.60 Bb	0.59 Bb	0.72 Ab	0.85 Ab	0.86 Ab	0.91 Ab
	KNO_3	0.23 Bb	0.33 Ab	0.43 Ab	0.52 Ab	0.57 Aa	0.66 Aa	0.72 Ab	0.81 Ab	0.86 Ab	0.97 Ab
	WN	0.19 Bb	0.31 Ab	0.42 Ab	0.49 Ab	0.54 Bb	0.58 Bb	0.64 Bb	0.65 Bc	0.73 Bb	0.71 Ab
Blue	$NaNO_3$	0.27 Aa	0.29 Ac	0.32 Ac	0.34 Ac	0.39 Bb	0.35 Bb	0.43 Ac	0.45 Bc	0.52 Ac	0.53 Ac
	KNO_3	0.23 Bb	0.29 Ac	0.32 Ac	0.36 Ac	0.42 Aa	0.44 Aa	0.46 Ac	0.52 Ac	0.50 Ad	0.57 Ac
	WN	0.21 Bb	0.26 Ac	0.31 Ac	0.35 Ac	0.38 Bb	0.39 Bb	0.44 Bc	0.37 Cd	0.47 Ac	0.46 Ad
Yellow	$NaNO_3$	0.27 Aa	0.38 Aa	0.55 Aa	0.61 Aa	0.71 Bb	0.75 Bb	0.92 Aa	0.90 Bb	1.12 Ba	1.22 Ba
	KNO_3	0.23 Bb	0.38 Aa	0.55 Aa	0.65 Aa	0.82 Aa	0.87 Aa	0.95 Aa	1.15 Aa	1.27 Aa	1.29 Aa
	WN	0.20 Bb	0.39 Aa	0.51 Aa	0.60 Aa	0.69 Bb	0.76 Bb	0.89 Ba	0.85 Ba	0.83 Cc	0.80 Ca
