# Supplementary material for: Optimal Laboratory Cultivation Conditions of Limnospira maxima for Large-Scale Production
Source: Biology (Basel). 2023 Nov 24;12(12):1462. doi: 10.3390/biology12121462 (PMC10740766; doi:10.3390/biology12121462)
Supplement: Supplementary file 1 [file biology-12-01462-s001.zip › Supplementary Table S2.pdf]

Supplementary Table S2. Effect of light spectra and nitrogen source on dry weight of *Limnospira maxima* cultures were grown under white, red, blue, and yellow light spectra, supplemented with $NaNO_3$, KNO_3, and a control (WN). The values presented in the figure indicate the means (± standard error). Capital letters indicate significant effects between nitrogen sources within the same light spectrum, while lowercase letters indicate significance between light spectra within the same nitrogen source. The statistical significance was determined using the SNK test.

Light spectra	N source	0	3	6	9	12	15	18	21	24	27
White	$NaNO_3$	0.27 Bc	0.46 Ba	0.56 Ba	0.63 Ca	0.81 Ba	0.88 Ca	1.02 Ca	1.02 Ca	1.25 Ba	1.27 Ba
	KNO_3	0.25 Bb	0.46 Ba	0.61 Ba	0.71 Ba	0.86 Ba	0.94 Ba	1.09 Ba	1.24 Ba	1.27 Ba	1.43 Aa
	WN	0.37 Aa	0.78 Aa	1.05 Aa	1.23 Aa	1.43 Aa	1.48 Aa	1.76 Aa	1.65 Aa	1.54 Aa	1.43 Aa
Red	$NaNO_3$	0.34 Ab	0.43 Aa	0.56 Ba	0.63 Ba	0.75 Bb	0.74 Bb	0.92 Bb	1.07 Ba	1.08 Ab	1.15 Ab
	KNO_3	0.24 Ab	0.35 Bb	0.46 Cc	0.55 Cc	0.60 Cc	0.70 Bc	0.76 Cc	0.86 Cc	0.92 Bb	1.03 Cc
	WN	0.29 Ab	0.48 Ab	0.65 Ac	0.76 Ac	0.83 Ac	0.89 Ac	0.99 Ac	1.00 Ac	1.12 Ac	1.09 Bc
Blue	$NaNO_3$	0.44 Aa	0.48 Aa	0.52 Ab	0.56 Bb	0.65 Bc	0.57 Bc	0.70 Bc	0.74 Bc	0.86 Ac	0.87 Bc
	KNO_3	0.38 Aa	0.49 Aa	0.55 Ab	0.61 Bb	0.71 Bb	0.74 Ab	0.78 Ac	0.88 Ac	0.84 Ac	0.97 Ad
	WN	0.40 Aa	0.50 Ab	0.59 Ad	0.69 Ad	0.75 Ac	0.76 Ad	0.86 Ad	0.72 Bd	0.92 Ad	0.89 Bd
Yellow	$NaNO_3$	0.26 Ac	0.37 Bb	0.54 Cb	0.60 Ba	0.70 Cbc	0.74 Cb	0.90 Cb	0.89 Cb	1.10 Cb	1.20 Aab
	KNO_3	0.23 Ab	0.39 Bb	0.56 Bb	0.66 Bb	0.83 Ba	0.89 Ba	0.96 Bb	1.17 Bb	1.29 Ba	1.31 Ab
	WN	0.34 Aa	0.65 Aa	0.84 Ab	0.99 Ab	1.15 Ab	1.27 Ab	1.48 Ab	1.41 Ab	1.38 Ab	1.33 Ab
