# Supplementary material for: Optimal Laboratory Cultivation Conditions of Limnospira maxima for Large-Scale Production
Source: Biology (Basel). 2023 Nov 24;12(12):1462. doi: 10.3390/biology12121462 (PMC10740766; doi:10.3390/biology12121462)
Supplement: Supplementary file 1 [file biology-12-01462-s001.zip › Supplementary Table S4.pdf]

Supplementary Table S4. Effect of light spectra and nitrogen source on total carotenoids concentration of *Limnospira maxima* grown in white, red, blue, and yellow light spectra, supplemented with $NaNO_3$, KNO_3, plus control (WN). Values followed by capital letter denote significative effect between N source in the same light spectra and small letter denote significance between light spectra in the same nitrogen source. All value represents media (± SD), followed by SNK statistical test.

Light spectra	N source	0	3	6	9	12	15	18	21	24	27
White	$NaNO_3$	0.91 Ab	1.51 Aa	1.87 Aa	2.09 Aa	2.67 Aa	2.91 Aa	3.40 Aa	3.39 Aa	4.14 Aa	4.22 Aa
	KNO_3	0.45 Cb	0.83 Bb	1.12 Cb	1.30 Bb	1.57 Bb	1.72 Ca	1.99 Ca	2.28 Ca	2.33 Ba	2.62 Ba
	WN	0.59 Bc	1.24 Ab	1.66 Bb	1.94 Ab	2.26 Ab	2.34 Ba	2.78 Ba	2.61 Bb	2.44 Bb	2.27 Bc
Red	$NaNO_3$	0.53 Ac	0.67 Ac	0.86 Bc	0.98 Bc	1.17 Bc	1.15 Bc	1.42 Bc	1.66 Ac	1.68 Bc	1.79 Bc
	KNO_3	0.50 Ab	0.73 Ac	0.95 Ac	1.14 Ac	1.26 Bc	1.45 Ab	1.59 Ab	1.78 Ab	1.90 Ac	2.13 Ac
	WN	0.49 Ad	0.82 Ac	1.10 Ac	1.28 Ac	1.40 Ac	1.51 Ab	1.67 Ab	1.70 Ad	1.91 Ac	1.85 Bd
Blue	$NaNO_3$	1.23 Aa	1.35 Ab	1.46 Bb	1.57 Bb	1.81 Bb	1.60 Cb	1.96 Bb	2.07 Bb	2.41 Bb	2.44 Bb
	KNO_3	0.97 Ca	1.23 Aa	1.38 Ba	1.55 Ba	1.80 Ba	1.88 Ba	1.97 Ba	2.23 Aa	2.14 Cb	2.44 Bb
	WN	1.35 Ba	1.66 Aa	1.99 Aa	2.30 Aa	2.50 Aa	2.55 Aa	2.87 Aa	2.42 Ac	3.07 Aa	2.99 Aa
Yellow	$NaNO_3$	0.20 Bd	0.29 Bd	0.42 Bd	0.46 Bd	0.54 Cd	0.57 Cd	0.69 Bd	0.68 Cd	0.85 Cd	0.92 Cd
	KNO_3	0.21 Bc	0.35 Bd	0.51 Bd	0.60 Bd	0.76 Bd	0.81 Bc	0.87 Bc	1.06 Bd	1.18 Bd	1.20 Bd
	WN	0.66 Ab	1.26 Ab	1.63 Ab	1.91 Ab	2.22 Ab	2.45 Aa	2.86 Aa	2.73 Aa	2.66 Ab	2.57 Ab

Supplementary Table S6. Impact of Light Spectra and Nitrogen Source on Phycocyanin Concentration in *Limnospira maxima* Cultivation. This figure showcases the influence of different light spectra and nitrogen sources on the concentration of phycocyanin in *Limnospira maxima* cultures. The cultures were supplemented with $NaNO_3$, KNO_3, or maintained as controls (WN) under white, red, blue, and yellow light spectra. The presented values denote the means along with their respective standard deviations (± SD). Capital letters indicate significant effects among nitrogen sources within the same light spectra, while lowercase letters denote significant differences among light spectra within the same nitrogen source. The statistical significance was determined through the SNK test.

Light spectra	N source	0	3	6	9	12	15	18	21	24	27
White	$NaNO_3$	81.00 Ab	134.37 Ab	166.48 Aa	185.97 Ba	237.57 Aa	259.15 Aa	302.30 Aa	301.68 Ba	368.18 Aa	375.69 Aa
	KNO_3	66.59 Bb	122.04 Bb	164.77 Aa	190.98 Aa	229.80 Aa	251.80 Aa	291.80 Ba	333.46 Aa	341.67 Ba	384.11 Aa
	WN	54.06 Bb	113.28 Cb	151.58 Ab	177.47 Cb	206.70 Ba	213.48 Bb	254.26 Ca	238.22 Ca	223.12 Cc	207.36 Bc
Red	$NaNO_3$	93.79 Ab	118.08 Ac	152.58 Aa	173.14 Abc	206.64 Abc	202.71 Ab	250.65 Ab	293.27 Aa	296.85 Ab	315.68 Ab
	KNO_3	59.17 Bb	86.48 Cc	112.39 Cc	135.49 Cc	149.24 Cc	172.52 Bc	188.71 Cc	211.73 Bd	225.91 Bc	253.39 Bc
	WN	61.49 Bb	101.58 Bc	137.56 Bc	159.93 Bc	174.62 Bc	188.24 Bc	207.91 Bc	211.47 Bb	237.19 Bb	230.71 Cb
Blue	$NaNO_3$	131.23 Aa	143.15 Aa	155.23 Ba	166.33 Bc	192.12 Bc	170.24 Cc	208.60 Bc	219.54 Bc	255.77 Bc	259.69 Ac
	KNO_3	102.99 Ba	131.22 Ca	146.48 Bb	164.79 Bb	191.49 Bb	199.58 Bb	209.19 Bb	237.11 Ac	226.89 Cc	252.61 Ac
	WN	116.17 Ba	143.29 Ba	171.54 Aa	198.85 Aa	215.50 Aa	220.36 Aa	247.49 Aa	208.95 Cb	265.07 Aa	258.15 Aa
Yellow	$NaNO_3$	71.56 Ac	102.57 Bc	148.42 Aa	163.43 Ac	191.57 Ac	202.72 Ab	247.49 Ab	242.72 Bb	301.69 Ab	328.66 Ab
	KNO_3	55.57 Bc	91.89 Cc	132.31 Bb	156.15 Ab	197.46 Ab	210.83 Ab	228.47 Bb	277.19 Ab	307.47 Ab	312.46 Ab
	WN	52.02 Bc	100.22 Bc	128.98 Cc	151.64 Ac	176.25 Bc	194.49 Ab	226.74 Bb	216.73 Cb	211.13 Bc	203.66 Bc
