# Supplementary material for: Optimal Laboratory Cultivation Conditions of Limnospira maxima for Large-Scale Production
Source: Biology (Basel). 2023 Nov 24;12(12):1462. doi: 10.3390/biology12121462 (PMC10740766; doi:10.3390/biology12121462)
Supplement: Supplementary file 1 [file biology-12-01462-s001.zip › Supplementary Table S6.pdf]

Supplementary Table S6. Effect of light spectra and nitrogen source on allophycocyanin concentration of *Limnospira maxima* grown in white, red, blue, and yellow light spectra, supplemented with $NaNO_3$, KNO_3, plus control (WN). Values followed by capital letters denote significant effects between N sources in the same light spectra, and small letters denote significance between light spectra in the same nitrogen source. All values represent means (± SD), followed by SNK statistical test.

Light spectra	N source	0	3	6	9	12	15	18	21	24	27
White	$NaNO_3$	34.58 Ab	57.31 Ab	71.08 Ab	79.40 Bb	101.43 Bb	110.64 Ab	129.07 Ab	128.80 Ab	157.19 Ab	160.40 Ab
	KNO_3	23.13 Bc	42.38 Bb	57.22 Bc	66.32 Cb	79.81 Cb	87.45 Bc	101.34 Bc	115.80 Ab	118.66 Bc	133.39 Bc
	WN	28.73 Bc	60.20 Ab	80.55 Ab	94.31 Ab	109.84 Ab	113.44 Ab	135.12 Ab	126.59 Ab	118.57 Bc	110.20 Cc
Red	$NaNO_3$	61.24 Aa	77.08 Aa	99.63 Aa	113.05 Aa	134.93 Aa	132.36 Aa	163.66 Aa	191.49 Aa	193.83 Aa	206.12 Aa
	KNO_3	33.60 Bb	49.10 Cb	63.81 Cb	76.93 Ca	84.73 Cb	97.95 Bb	107.15 Bab	120.22 Bb	128.27 Bb	143.87 Bb
	WN	33.34 Bbc	55.08 Bb	74.59 Bb	86.72 Bbc	94.69 Bc	102.08 Bc	112.74 Bc	114.68 Bc	128.62 Bb	125.11 Cb
Blue	$NaNO_3$	64.59 Ba	70.45 Ba	76.40 Bb	81.86 Bb	94.56 Bc	83.79 Cc	102.67 Bc	108.05 Bc	125.89 Bd	127.81 Bc
	KNO_3	50.69 Ca	64.58 Ba	72.09 Ba	81.10 Ba	94.25 Ba	98.23 Bb	102.96 Bb	116.70 Bb	111.67 Cc	127.81 Bc
	WN	74.59 Aa	92.00 Aa	110.14 Aa	127.67 Aa	138.36 Aa	141.48 Aa	158.90 Aa	134.16 Aa	170.19 Aa	165.75 Aa
Yellow	$NaNO_3$	30.03 Ab	43.04 Bc	62.28 Ac	68.58 Bc	80.39 Bd	85.06 Bc	103.85 Cc	101.85 Cd	126.60 Bc	137.91 Bc
	KNO_3	27.19 Ac	44.96 Bb	64.74 Aab	76.40 Aa	96.61 Aa	103.15 Aa	111.78 Ba	135.62 Aa	150.43 Aa	152.88 Aa
	WN	28.76 Ac	55.41 Ab	71.32 Ab	83.85 Ac	97.45 Ac	107.54 Ac	125.37 Ab	119.83 Bbc	116.74 Bc	112.61 Cc
