# Supplementary material for: Optimal Laboratory Cultivation Conditions of Limnospira maxima for Large-Scale Production
Source: Biology (Basel). 2023 Nov 24;12(12):1462. doi: 10.3390/biology12121462 (PMC10740766; doi:10.3390/biology12121462)
Supplement: Supplementary file 1 [file biology-12-01462-s001.zip › Supplementary Table S7.pdf]

Supplementary Table S7. Effect of light spectra and nitrogen source on phycoerytrin concentration of *Limnospira maxima* grown in white, red, blue, and yellow light spectra, supplemented with $NaNO_3$, KNO_3, plus control (WN). Values followed by capital letter denote significative effect between N source in the same light spectra and small letter denote significance between light spectra in the same nitrogen source. All value represents media (± SD), followed by SNK statistical test.

Light spectra	N source	0	3	6	9	12	15	18	21	24	27
White	$NaNO_3$	17.38 Ac	28.83 Ab	35.71 Ab	39.89 Bb	50.96 Ab	55.59 Ab	64.85 Aa	64.72 Ab	78.98 Ab	80.60 Ab
	KNO_3	9.81 Cc	17.98 Bc	24.28 Bc	28.14 Cc	33.86 Cb	37.11 Cc	43.00 Cc	49.14 Cc	50.35 Bb	56.60 Bb
	WN	12.94 Bc	27.12 Ab	36.29 Ab	42.49 Ab	49.48 Ba	51.11 Ba	60.87 Ba	57.03 Ba	53.42 Bb	49.64 Cc
Red	$NaNO_3$	25.75 Ab	32.41 Aa	41.90 Aa	47.54 Aa	56.74 Aa	55.66 Aa	68.82 Aa	80.52 Aa	81.51 Aa	86.68 Aa
	KNO_3	13.55 Bb	19.80 Cbc	25.73 Cc	31.02 Cb	34.17 Cb	39.50 Cc	43.21 Cc	48.48 Cc	51.72 Cb	58.02 Bb
	WN	16.11 Bb	26.61 Bb	36.04 Bb	41.90 Bb	45.75 Ba	49.31 Ba	54.47 Bc	55.40 Ba	62.14 Ba	60.44 Ba
Blue	$NaNO_3$	31.28 Aa	34.12 Aa	37.00 Ab	39.65 Bb	45.80 Ac	40.58 Cc	49.73 Bb	52.33 Bc	60.97 Ac	61.91 Ac
	KNO_3	24.55 Ba	31.28 Aa	34.92 Aa	39.28 Ba	45.65 Aa	47.58 Bb	49.89 Bb	56.52 Ab	54.09 Bb	60.93 Bb
	WN	26.97 Ba	33.26 Aa	39.82 Aa	46.16 Aa	50.03 Aa	51.15 Aa	57.45 Ac	48.51 Cb	61.53 Aa	59.93 Ba
Yellow	$NaNO_3$	11.54 Ad	16.55 Cc	23.94 Bc	26.36 Cc	30.90 Bd	32.70 Cd	39.92 Bc	39.15 Cd	48.67 Cd	53.02 Bd
	KNO_3	13.63 Ab	22.53 Bb	32.44 Ab	38.29 Ba	48.42 Aa	51.70 Aa	56.02 Aa	67.97 Aa	75.39 Aa	76.62 Aa
	WN	13.65 Ac	26.29 Ab	33.84 Ab	39.78 Ac	46.24 Aa	51.03 Ba	59.49 Ab	56.86 Ba	55.39 Bb	53.43 Bb
