# Supplementary material for: Optimal Laboratory Cultivation Conditions of Limnospira maxima for Large-Scale Production
Source: Biology (Basel). 2023 Nov 24;12(12):1462. doi: 10.3390/biology12121462 (PMC10740766; doi:10.3390/biology12121462)
Supplement: Supplementary file 1 [file biology-12-01462-s001.zip › Supplementary Table S8.pdf]

Supplementary Table S8. Effect of light spectra and nitrogen source on soluble proteins concentration of *Limnospira maxima* grown in white, red, blue, and yellow light spectra, supplemented with $NaNO_3$, KNO_3, plus control (WN). Values followed by a capital letter denote a significant effect between N source in the same light spectra, and a small letter denotes significance between light spectra in the same nitrogen source. All values represent the mean (± SD), followed by the SNK statistical test.

Light spectra	N source	0	3	6	9	12	15	18	21	24	27
White	$NaNO_3$	26.45 Ab	43.87 Ba	54.36 Ba	60.72 Ba	77.57 Ba	84.62 Ba	98.71 Ba	98.50 Ba	120.22 Aa	122.67 Aa
	KNO_3	17.47 Bb	32.01 Cb	43.22 Cb	50.10 Cb	60.28 Cb	66.05 Cb	76.54 Cb	87.46 Cb	89.62 Bb	100.75 Cb
	WN	28.44 Ab	59.58 Ab	79.73 Ab	93.35 Ab	108.72 Ab	112.29 Ab	133.74 Ab	125.30 Ab	117.36 Ab	109.07 Bb
Red	$NaNO_3$	31.06 Ab	39.09 Aa	50.52 Aa	57.33 Aa	68.42 Ab	67.12 Ab	83.00 Ab	97.11 Aa	98.30 Ab	104.53 Ab
	KNO_3	22.32 Ba	32.62 Bb	42.39 Bb	51.11 Ab	56.29 Bc	65.08 Ab	71.19 Bc	79.87 Bc	85.22 Bb	95.58 Bb
	WN	21.30 Bc	35.19 Bd	47.65 Ac	55.40 Ac	60.49 Bc	65.20 Ac	72.02 Bc	73.25 Bc	82.16 Bc	79.91 Cc
Blue	$NaNO_3$	36.38 Aa	39.68 Aa	43.03 Bb	46.11 Bb	53.26 Bc	47.19 Cc	57.83 Bc	60.86 Bc	70.90 Ac	71.99 Ac
	KNO_3	28.55 Ba	36.37 Ab	40.60 Bb	45.68 Bc	53.09 Bc	55.32 Bc	57.99 Bd	65.73 Ad	62.89 Cc	72.03 Ac
	WN	33.92 Ab	41.84 Ac	50.09 Ac	58.06 Ac	62.93 Ac	64.34 Ac	72.26 Ac	61.01 Bd	77.40 Ad	75.38 Ac
Yellow	$NaNO_3$	23.52 Bb	33.72 Cb	48.79 Ca	53.72 Cb	62.97 Cb	66.64 Cb	81.35 Cb	79.79 Cb	99.17 Cb	108.04 Cb
	KNO_3	25.30 Ba	41.83 Ba	60.23 Ba	71.09 Ba	89.89 Ba	95.98 Ba	104.01 Ba	126.19 Ba	139.97 Ba	142.24 Ba
	WN	39.04 Aa	75.22 Aa	96.81 Aa	113.82 Aa	132.29 Aa	145.98 Aa	170.18 Aa	162.67 Aa	158.46 Aa	152.86 Aa
