# Supplementary figures and images for: Optimal Laboratory Cultivation Conditions of Limnospira maxima for Large-Scale Production
Source: Biology (Basel). 2023 Nov 24;12(12):1462. doi: 10.3390/biology12121462 (PMC10740766; doi:10.3390/biology12121462)

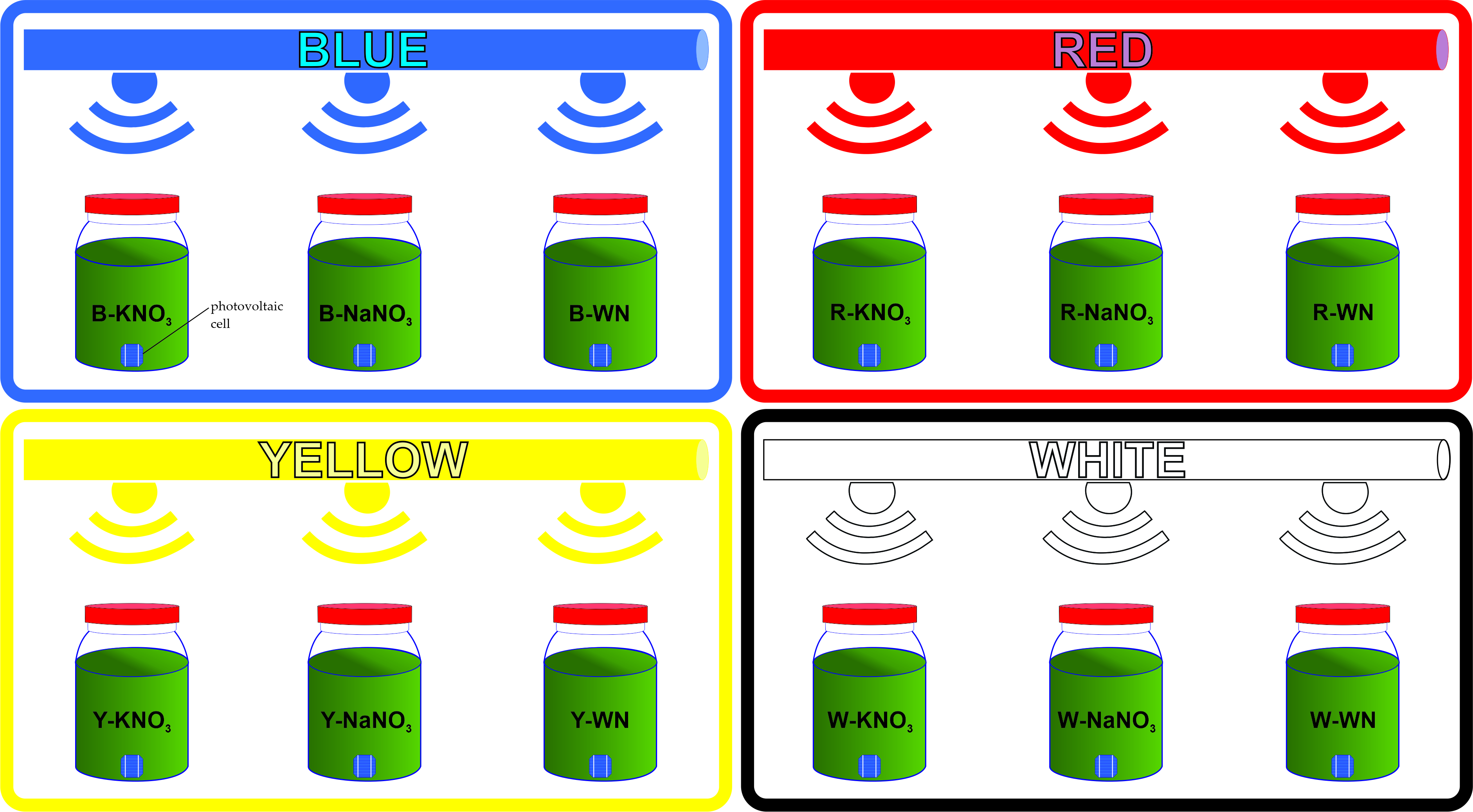

Supplement: Supplementary file 1 [file biology-12-01462-s001.zip › Supplementary Figure S1.jpg]
